# Understanding the effect of thickness on the thermoelectric properties of Ca_3_Co_4_O_9_ thin films

**DOI:** 10.1038/s41598-021-85287-2

**Published:** 2021-03-18

**Authors:** Yinong Yin, Ashutosh Tiwari

**Affiliations:** Nanostructured Materials Research Laboratory, Department of Material Science and Engineering, University of Utah, Salt Lake City, UT 84112 USA

**Keywords:** Electronic properties and materials, Thermoelectrics, Surfaces, interfaces and thin films

## Abstract

We are reporting the effect of thickness on the Seebeck coefficient, electrical conductivity and power factor of Ca_3_Co_4_O_9_ thin films grown on single-crystal Sapphire (0001) substrate. Pulsed laser deposition (PLD) technique was employed to deposit Ca_3_Co_4_O_9_ films with precisely controlled thickness values ranging from 15 to 75 nm. Structural characterization performed by scanning electron microscopy (SEM) and atomic force microscopy (AFM) showed that the growth of Ca_3_Co_4_O_9_ on Sapphire (0001) follows the island growth-mode. It was observed that in-plane grain sizes decrease from 126 to 31 nm as the thickness of the films decreases from 75 to 15 nm. The thermoelectric power measurements showed an overall increase in the value of the Seebeck coefficient as the films’ thickness decreased. The above increase in the Seebeck coefficient was accompanied with a simultaneous decrease in the electrical conductivity of the thinner films due to enhanced scattering of the charge carriers at the grain boundaries. Because of the competing mechanisms of the thickness dependence of Seebeck coefficient and electrical conductivity, the power factor of the films showed a non-monotonous functional dependence on thickness. The films with the intermediate thickness (60 nm) showed the highest power factor (~ 0.27 mW/m-K^2^ at 720 K).

## Introduction

Thermoelectricity has great potential for application in vibration-less thermoelectric power generators, solid-state coolers as well as embedded hot-spot cooling systems in next-generation power-efficient electronic devices^1–3^. The efficiency of a thermoelectric (TE) system depends on a dimensionless quantity known as the figure of merit, *ZT*, which depends on the temperature (T), Seebeck coefficient (*S*), electrical conductivity (*σ*) and thermal conductivity (*κ*) of the material by the relation: ZT = S^2^*σ*T/ *κ*^1^.

Ca_3_Co_4_O_9_ (CCO) is a promising TE material with reasonably good ZT values and several additional advantageous features such as high-temperature stability and non-toxicity. It possesses a layered structure, where two sublattices of CoO_2_ and Ca_3_CoO_2_ are stacked together along *c*-axis^4^. The conducting layers of CoO_2_ are responsible for the transport of charge carriers, while the insulating layers of Ca_3_CoO_2_ act as charge reservoirs. Such naturally occurring superlattice structure of CCO and misfit interfaces present between its two sublattices give rise to high *S*, moderate *σ* and low *κ*, which yield a reasonably high *ZT* value of around 0.87 at 973 K in its single-crystal form^5^. High ZT values have also been reported for polycrystalline CCO on doping small amount of rare earth elements in the system^6–16^.

Recently thin film thermoelectrics has attracted lots of attention because of its potential for application in next-generation electronic devices^17–22^. As the Moore’s law is approaching towards its saturation, the design of electronic devices is facing continuously escalating problems for thermal management. Specifically, localized regions of large heat flux on semiconductor chips give rise to hot spots that creates lifetime and reliability problems for devices^2^. Bulk thermoelectric coolers cannot be employed for such applications and hence it is necessary to develop thin film thermoelectric systems that can be embedded in integrated circuits.

Inspired by the above prospects, in this study we have explored the growth and thickness dependence of the thermoelectric properties of CCO thin films deposited by PLD technique on Sapphire (001) substrate. The study was further motivated by earlier reports that showed the growth of CCO on Sapphire obeys the island-growth mode, resulting in rough and grainy surface morphologies of the films^23,24^. This suggests that because of the energy filtering effects at grain boundaries, the CCO films can exhibit an enhanced Seebeck coefficient compared to bulk counterpart. Such improvement in Seebeck coefficient has indeed been demonstrated by P. Brinks et al*.* in the case of Na_x_CoO_2_ thin films that possess similar crystal structure as CCO^25^.

## Experimental details

Polycrystalline CCO target employed in PLD deposition was prepared using a solid-state process^26^. For this, fine powders of Calcium oxide (CaO, Alfa Aesar 99.9%) and Cobalt oxide (Co_3_O_4_, Alfa Aesar, 99.7%) were mixed together in stoichiometric ratios followed by a fine grinding to produce homogeneous mixture. This homogenous mixture was calcined twice with intermediate grinding steps. The calcined powder was pressed into a one-inch-diameter pellet using a uniaxial press and then was subjected to a cold isostatic pressure of ~ 205 MPa for few minutes. The resulting pellet was sintered at 900 °C to produce a compact and hard CCO target suitable for PLD ablation. The CCO target was mounted on a sample holder inside the PLD chamber. The deposition chamber was first pumped down to a base pressure of ~ 1 × 10^–6^ Torr and then high purity oxygen gas was introduced in the chamber to maintain a pressure of 150 mTorr during the deposition. Laser pulses from a KrF excimer laser (wavelength = 248 nm, pulse-width = 25 ns) were irradiated over the CCO target and the ablated material was deposited on the Sapphire (0001) substrate. During the deposition, substrate’s temperature was maintained at 600 °C. Energy density and repetition rate of the laser pulses used for ablation were ~ 1.2 J/cm^2^ and 3 Hz, respectively. Thickness of the films was controlled by varying the number of laser pulses. Films were grown with 2000, 4000, 6000, 8000 and 10,000 pulses and were named as CCO 2000, CCO 4000, CCO 6000, CCO 8000 and CCO 10,000, respectively. After deposition, films were annealed for 30 min in an oxygen atmosphere at a pressure of 750 mTorr followed by cooling to room temperature with a cooling rate of 5 °C /min.

The *θ*–2*θ* X-ray diffraction (XRD) patterns of the samples were recorded using Panalytical X-Pert X-Ray diffractometer to determine the crystal structure of the films. In order to look into the atomic scale arrangement inside the crystal structure, high-resolution transmission electron microscopy (TEM) was performed on the CCO 8000 sample using a JEOL JEM 2800 transmission electron microscope. The surface morphology and roughness of the films was determined using a Bruker Dimension ICON-PT atomic force microscope (AFM) and analyzed using NanoScope Analysis software NanoScope Analysis software (Version:1.4; http://nanoscaleworld.bruker-axs.com/nanoscaleworld/forums/t/812.aspx). The microstructural characterizations of the surfaces were performed using FEI Quanta 600 FEG scanning electron microscope (SEM), which provided crucial information about the in-plane grain sizes present in the films. To determine the growth rate, the thickness of CCO 8000 was measured by performing the cross-sectional SEM of the film. The above thickness was found to be 60 nm, which gave a growth rate of 0.075 Å /pulse. Using this value of growth rate, number of laser pulses were varied to get the films of different thicknesses. Exact values of the thicknesses of individual films were determined by SEM and were found to be a linear function of the number of pulses used for deposition.

The electrical conductivity of the samples was measured by conventional four-probe method over the temperature range of 440–720 K. Seebeck coefficient measurements were performed over the temperature range of 440–720 K. At each measurement point, a temperature difference was created across the film and the resulting Seebeck voltage was measured using a Keithley 181 Nanovoltmeter. Temperatures were measured via two K-type thermocouples connected to the two ends of the film. The power factors (PF = *S*^*2*^*σ*) for all the films were calculated by using the experimentally measured values of Seebeck coefficient and electrical conductivity.

## Results and discussion

### Structural properties

**Figure 1:**
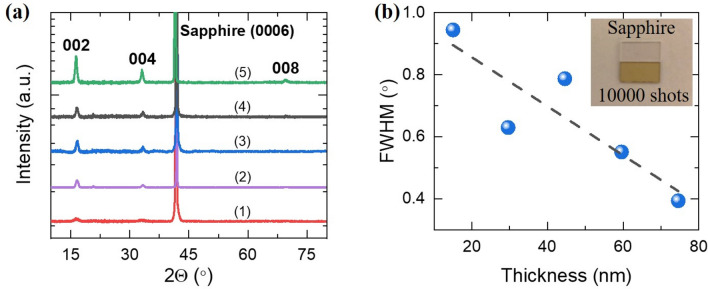
**(a)** The X-ray diffraction patterns for the Ca_3_Co_4_O_9_ (CCO) thin films. (1)–(5) represents the films with thickness of 15 nm, 30 nm, 45 nm, 60 nm and 75 nm, respectively; **(b)** FWHM of (002) peaks for the CCO films with different thicknesses. The inset shows an optical photograph of CCO 10,000 film on Sapphire substrate along with that of the bare Sapphire substrate.

Figure 1a shows the XRD patterns recorded from the thin film samples. Apart from the (0006) peak of the sapphire substrate, 3 additional peaks were observed at around 2*θ* values of 16.3°, 33.0° and 69.4°. These peaks were found to correspond to the (002), (004), (008) planes of Ca_3_Co_4_O_9_, indicating *c*-axis preferred textured growth of films. In Fig. 1b we have shown the full-width at half maximum (FWHM) of the (002) peaks as a function of the thickness of the films. A decrease in the values of FWHM with thickness can clearly be seen. Since the FWHM of the peaks is inversely proportional to the crystallite size^27^, it is inferred that the overall crystallite size in the film increases with increase in the thickness of the film. The inset of Fig. 1b displays a typical optical image of the CCO 10,000 film showing its semi-transparent nature. For comparison an optical image of the bare sapphire substrate has also been shown.

**Figure 2:**
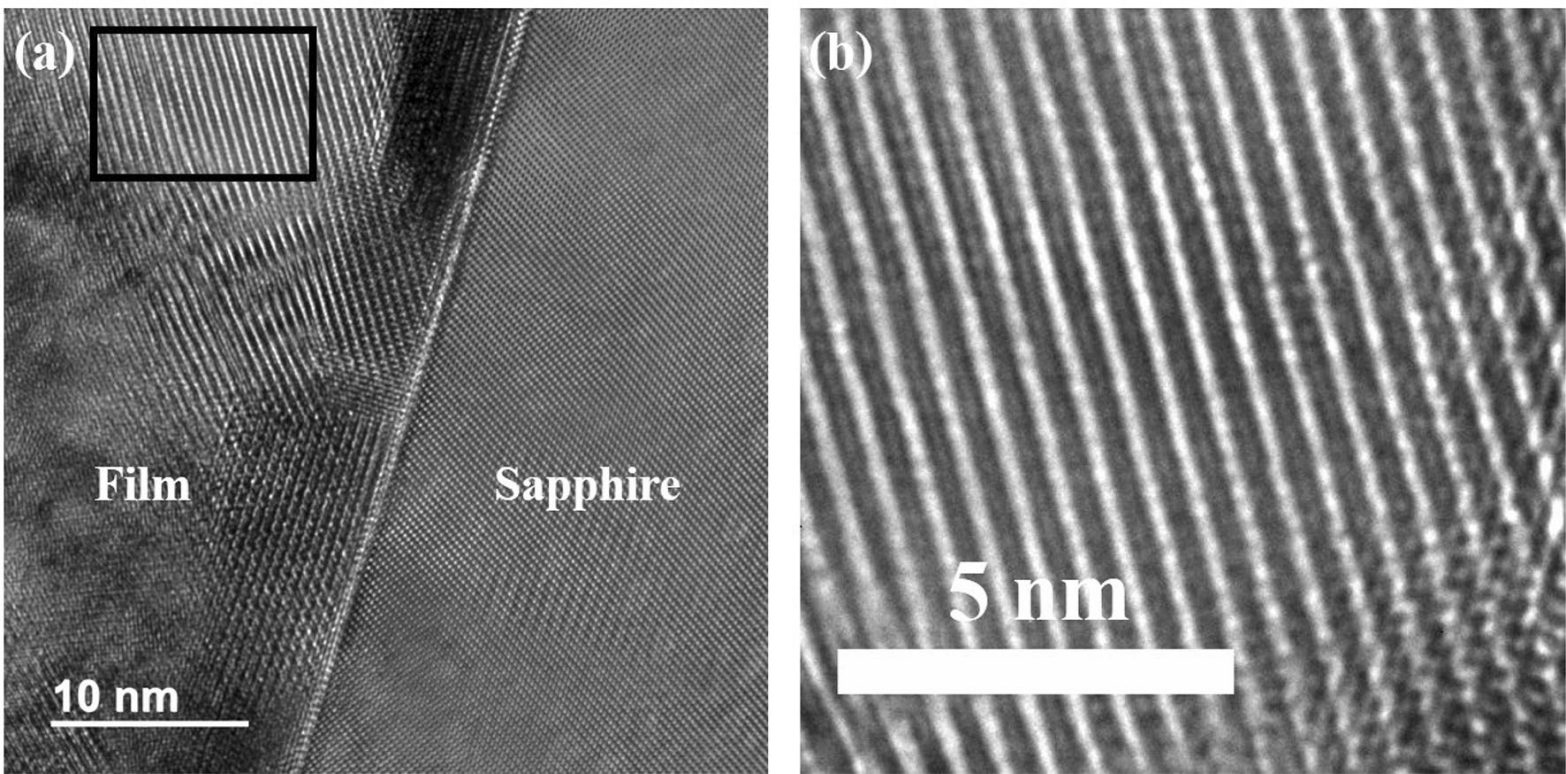
**(a)** Cross-sectional transmission electron micrograph at the interface of CCO 8000 film and substrate. The magnification of the marked area in **(a)** is shown in **(b),** where sublattice layers stacked alternately along *c*-axis can clearly be seen.

In Fig. 2a we have shown a typical high-resolution TEM image obtained from the interface of the CCO 8000 film and the substrate. Figure 2b shows the zoomed-in image of the section marked in Fig. 2a. From these images, the layered structure of the film can clearly be envisaged. The distance between the consecutive layers was found to 0.55 nm, which is half the reported value of the inter-planar separation between CoO_2_ layers in Ca_3_Co_4_O_9_^28,29^.

**Figure 3:**
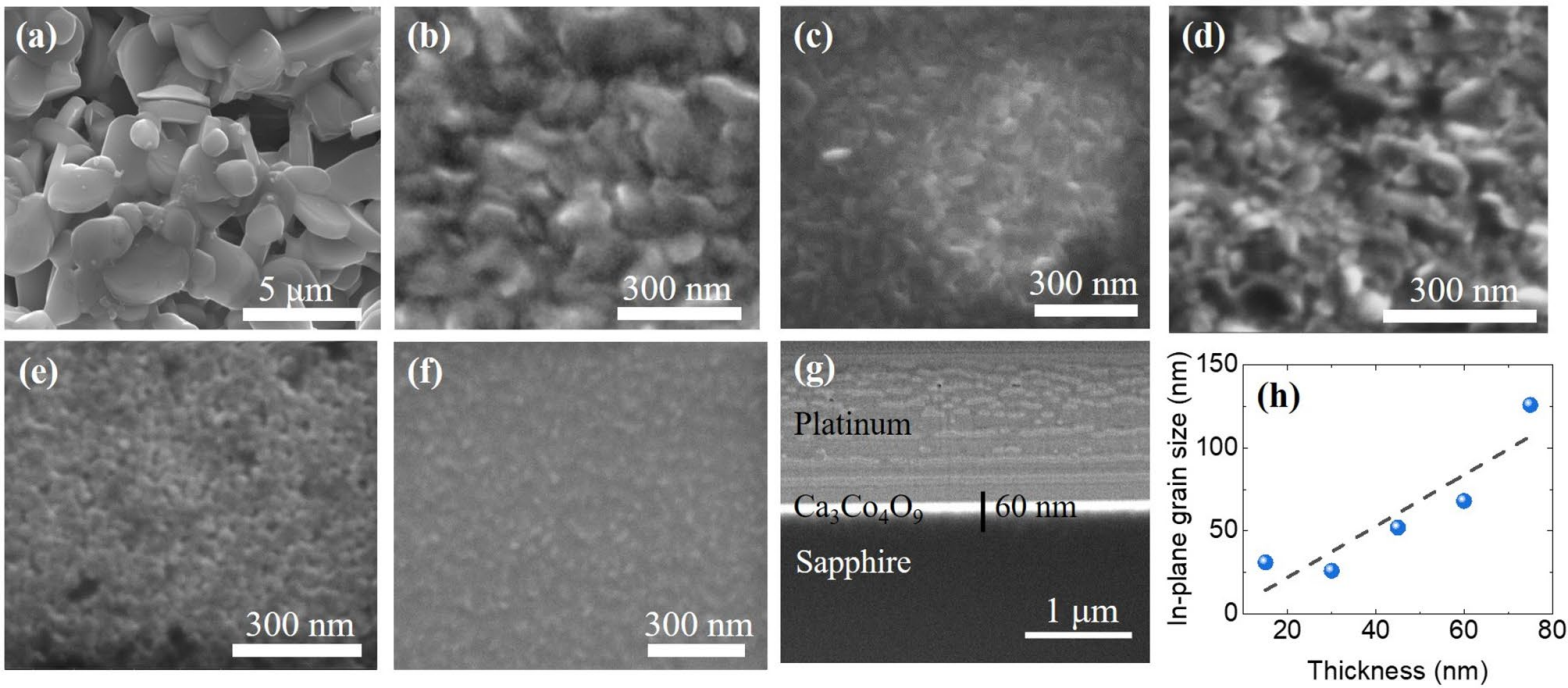
The top-view SEM images of **(a)** bulk Ca_3_Co_4_O_9_ and thin films with thickness of **(b)** 75 nm, **(c)** 60 nm, **(d)** 45 nm, **(e)** 30 nm and **(f)** 15 nm. The cross-section SEM of interfacial region **(g)** the thickness of Ca_3_Co_4_O_9_ films prepared with 8000 laser shots is around 60 nm. **(h)** Variation of in-plane grain sizes in various CCO films as a function of thickness.

Figure 3 shows the SEM images recorded from various CCO thin films. For comparison, SEM image recorded from the bulk pellet has also been shown. As can be seen, while the bulk pellet consists of micron-sized grains, the films are composed of nano-sized grains and are much denser than the bulk pellet. The average in-plane grain sizes for individual films were estimated using the ImageJ software (Version: IJ1 ; https://imagej.net/ImageJ)^30^. It was found that the grain size decreases from ~ 126 nm to ~ 31 nm as the thickness of films decreases from 75 to 15 nm (See Fig. 3h). This finding is in good agreement with the variation of FWHM of XRD peaks of the films with thicknesses.

**Figure 4:**
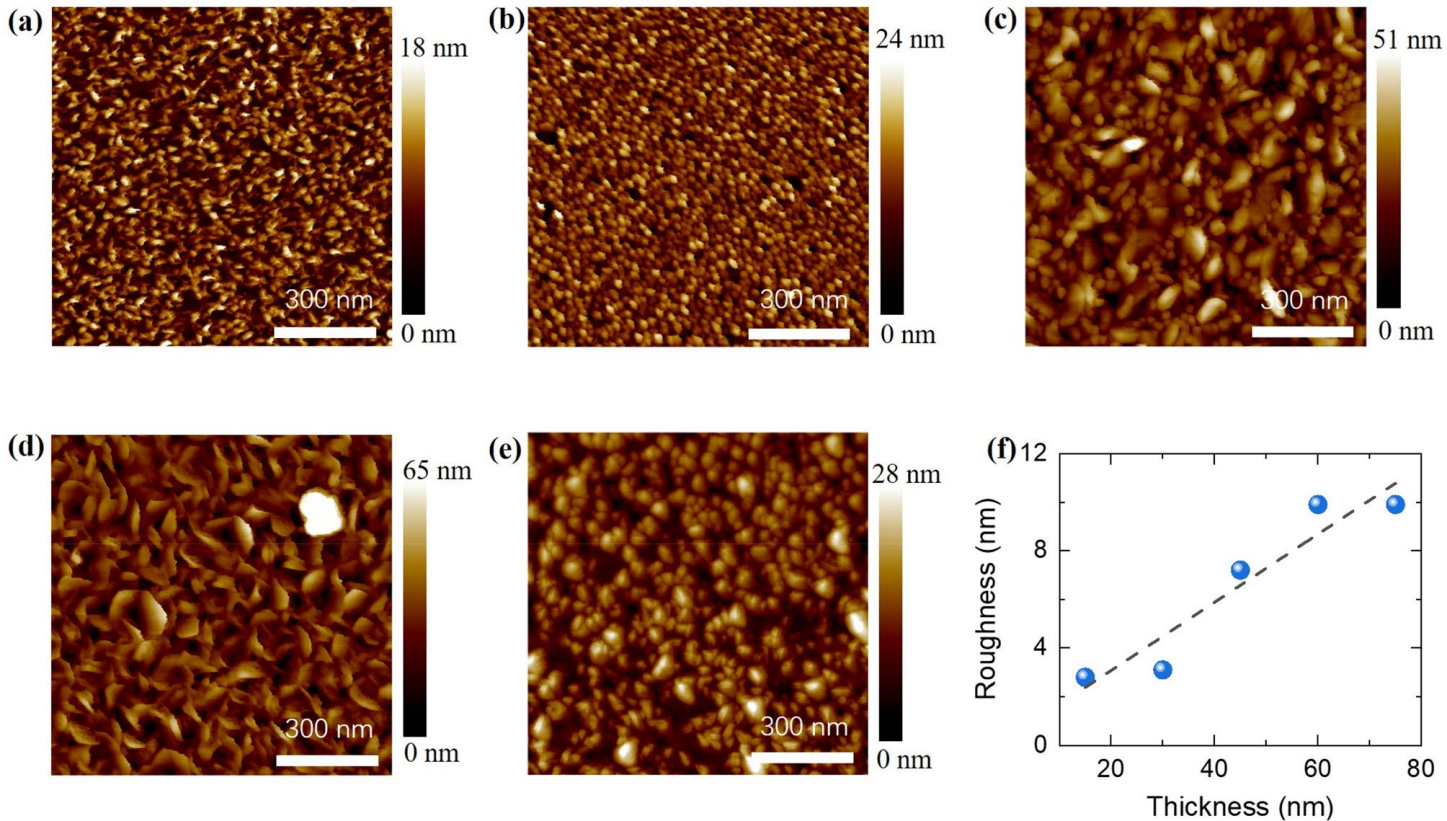
AFM phase images of the CCO films with thickness of **(a)** 15 nm, **(b)** 30 nm, **(c)** 45 nm, **(d)** 60 nm and **(e)** 75 nm; **(f)** shows the variation of films’ roughness with thickness.

Figure 4 shows the AFM images of the CCO thin films. As can be clearly seen, all the films possess grainy surface microstructure with root-mean-square roughness (*Rq*) ranging from ~ 2.8 to ~ 9.9 nm. In Fig. 4f we have plotted the roughness in CCO films as a function of film’s thickness. The observed topological characteristics of our films are quite similar as reported by other groups where they also observed the island-growth mode of CCO on sapphire substrate^23,24^.

### Thermoelectric properties

**Figure 5:**
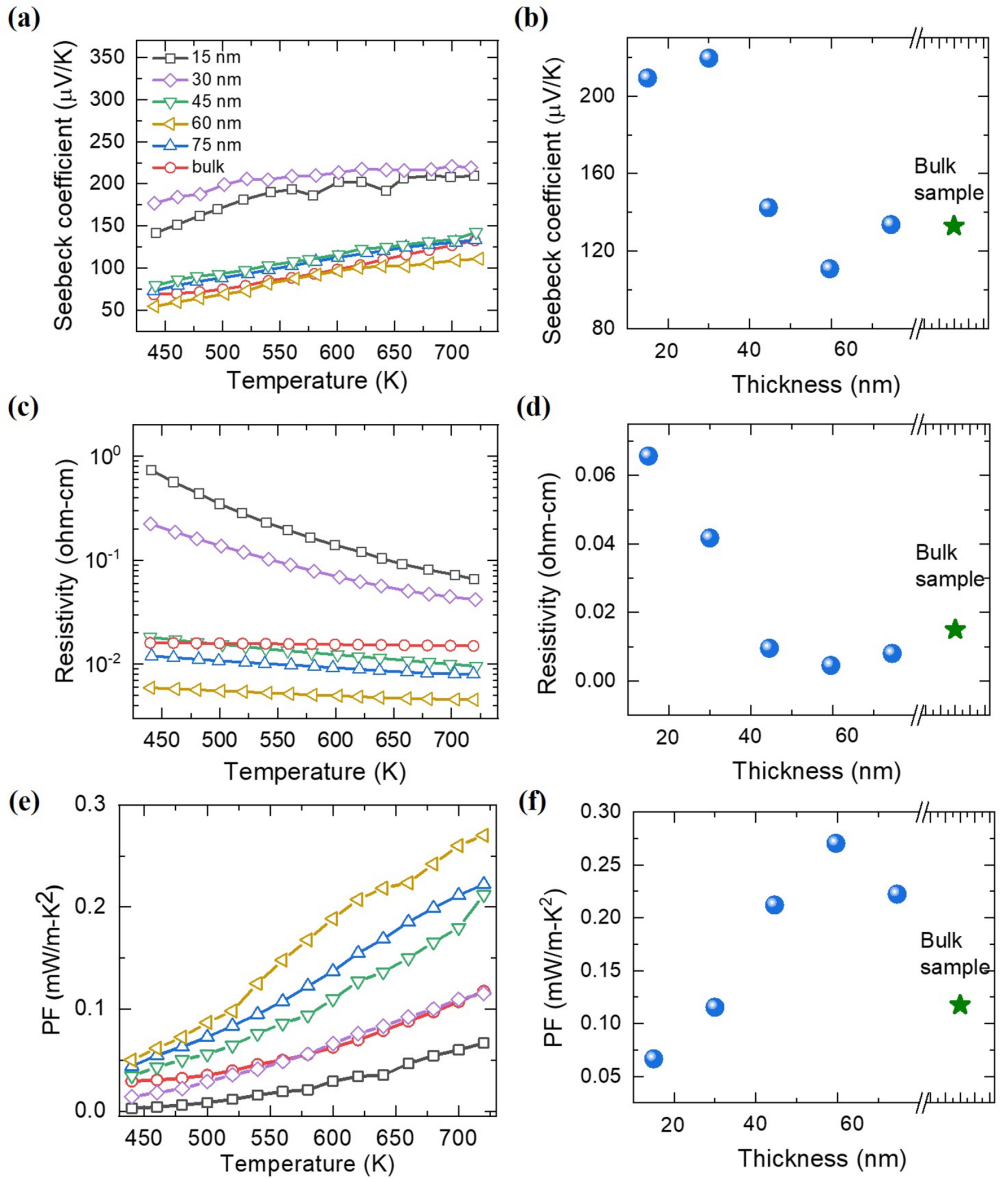
**(a**,**c**,**e)** The Seebeck coefficient, (*S*), resistivity (*ρ*), and power factors of various thin films and bulk CCO as a function of temperature (*T*); **(b**,**d**,**f)** show the comparisons of the *S*, *ρ*, and power factors at 720 K of CCO thin films and bulk CCO.

Figure 5a shows the Seebeck coefficient of CCO thin films measured over the temperature range of 440–720 K. For comparison we have also shown the corresponding data for the bulk sample. It can be seen that *S* shows an increase with increase in temperature. Such temperature dependence is consistent with the predictions from the small polaron theory where the Seebeck coefficient is given by the expression^1^:

1 $$S=\frac{{k}_{B}}{e}(\gamma {k}_{B}T+\frac{\Delta {S}^{*}}{{k}_{B}}),$$

where *k*_*B*_ is the Boltzmann constant, ΔS* is the average change in the entropy of the material upon injection of a charge carrier. The term (*γk*_*B*_*T*) is a dimensionless parameter linearly proportional to temperature, where ***γ*** is a constant defined by *γ* = *zJ*^*2*^*/2* ${E}_{S}^{3}$ (*z* is the number of nearest neighbors for the hopping site, *J* is the inter-site transfer energy and *E*_*S*_ is the small polaron’s binding energy). The second term of ΔS*/*k*_*B*_ refers to an intrinsic material property determined by carrier concentrations and is almost independent of temperature for a degenerate semiconductor with fixed carrier concentration. Hence, according to this formula, the value of *S* should be linearly proportional to *T* for the materials where conduction takes place by the hopping of small polarons. As shown in Fig. 5a, the Seebeck coefficients of all CCO samples indeed increase almost linearly with temperature, in good agreement with the predictions of the small-polaron theory. Another important point to be noticed is that the thinner films show the larger Seebeck coefficient values compared to thicker films. Figure 5b shows a direct comparison of Seebeck coefficients of different films and the bulk sample at 720 K. As is evident, overall value of the Seebeck coefficient increases as the thickness of the films decreases. The reason responsible for the thermopower enhancement in the thinner films can be attributed to the energy filtering effect. Specifically, since *S* relies on the average energy of charge carriers relative to the *E*_*F*_^1^, filtering out low-energy carriers due to the presence of energy barriers at grain boundaries is advantageous for the improvement of *S*. The results of SEM and AFM indicated that the grain sizes in our films decrease as the thickness goes down. Therefore, increased number of the carriers with low energies can be trapped at grain boundaries in thinner films resulting in higher Seebeck coefficient values compared to thicker films and bulk sample. Such effect of grain size on *S* has been reported in many other nanocomposite^31^ and thin film^25^ systems as well.

In Fig. 5c, we have shown the temperature dependent electrical resistivity of various CCO thin films and the bulk sample. Temperature dependence of the electrical resistivity data was also found to obey the small-polaron hopping conduction mechanism. As per the small polaron theory, the relation between electrical conductivity (*σ*) and *T* is given by the expression^1^:

2 $$\sigma =\frac{c(1-c){e}^{2}}{a{k}_{B}T}{\tau }_{0}^{-1}\mathrm{exp}(-\frac{{E}_{\sigma }}{{k}_{B}T}),$$

where *c* is the fractional concentration of charge carriers, *a* is the jump distance of the carriers, *τ*_*0*_ is a constant, and *E*_*σ*_ is the activation energy that corresponds to the work needed by the lattice to adjust so that the site at which the charge is positioned and that to which it jumps are degenerate. By fitting the experimental data to the above expression the values of *E*_*σ*_ for the films with thickness values of 15 nm, 30 nm, 45 nm, 60 nm and 75 nm were found to be 0.124 (± 0.001) eV, 0.095 (± 0.001) eV, 0.048 (± 0.001) eV, 0.033 (± 0.001) eV and 0.039 (± 0.001) eV, respectively. The observed ascending values of *E*_*σ*_ as the thickness declines, suggest that more thermal energies are required to enable the transport of polarons in thinner films. Furthermore, on comparing the resistivity at 720 K of various CCO thin films, it was noticed that the resistivity of the 15 nm thick film is almost 5 times as much as the resistivity of the bulk CCO pellet (see Fig. 5d). These findings are consistent with the AFM results, which showed that thinner films exhibit smaller grains and hence more grain boundaries compared to the thicker films and the bulk pellet. Increased density of grain boundaries immobilize free charge carriers and weaken electrical conductivity of the films.

Figure 5e shows the power factors (S^2^σ*)* of CCO samples obtained from the experimentally measured values of *S* and *σ*. For all the samples, power factor shows an increase with increase in the temperature. For the sake of comparison, in Fig. 5f, we have plotted the power factor of various samples at 720 K. It is interesting to note that, despite its relatively large Seebeck coefficient, the 15 nm thick CCO film shows the lowest power factor due to its low electrical conductivity. However, when the thickness of the films increases, the power factor first increases and then starts decreasing after reaching a maximum value for the intermediate thickness. The film with the thickness value of 60 nm showed the highest power factor (~ 0.27 mW/m-K^2^ at 720 K) among all the samples.

## Conclusion

In summary, we have gained a thorough understanding of how the thickness of Ca_3_Co_4_O_9_ films affects the underlying processes responsible for the thermoelectric performance of the material. Ca_3_Co_4_O_9_ films with a range of thicknesses were prepared using PLD technique. It was found that the growth of Ca_3_Co_4_O_9_ films on sapphire (0001) substrate follows the island growth mode. A relationship between the in-plane grain sizes and the thickness of the films was observed, showing a descending trend of the grain sizes with decrease in the thickness of the films. Both, the Seebeck coefficient as well as the electrical conductivity of the films was found to obey the small polaron hopping mechanism. Because of the enhanced energy filtering effect, Seebeck coefficient for thinner films was found to be significantly higher than that for thicker ones. However, thinner films also exhibited higher activation energies for electrical conduction resulting in relatively lower electrical conductivities than thicker films. Because of the opposite thickness dependences of Seebeck coefficient and electrical conductivity, the films with intermediate thickness were found to exhibit the highest power factor. Optimization of the thermoelectric response of CCO by just controlling the thickness of the films is interesting and has potential to lead to efficient thin film thermoelectric devices and embedded coolers in future.
